# Persistent organic pollutants, pre-pregnancy use of combined oral contraceptives, age, and time-to-pregnancy in the SELMA cohort

**DOI:** 10.1186/s12940-020-00608-8

**Published:** 2020-06-15

**Authors:** Richelle D. Björvang, Chris Gennings, Ping-I Lin, Ghada Hussein, Hannu Kiviranta, Panu Rantakokko, Päivi Ruokojärvi, Christian H. Lindh, Pauliina Damdimopoulou, Carl-Gustaf Bornehag

**Affiliations:** 1 Division of Obstetrics and Gynecology, Department of Clinical Science, Intervention and Technology, Karolinska Institutet and Karolinska University Hospital, Stockholm, Sweden; 2 Unit of Toxicology Sciences, Swetox, Karolinska Institute, Södertälje, Sweden; 3 Department of Environmental Medicine and Public Health, Icahn School of Medicine at Mount Sinai, New York, USA; 4 Department of Health Sciences, Karlstad University, Karlstad, Sweden; 5 Department of Obstetrics and Gynecology, Karlstad Central Hospital, Karlstad, Sweden; 6 Department of Health Security, National Institute for Health and Welfare, Kuopio, Finland; 7 Department of Laboratory Medicine, Division of Occupational and Environmental Medicine, Lund, Sweden

**Keywords:** Persistent organic pollutants, Combined oral contraceptives, Time-to-pregnancy, Fecundability, Polychlorobiphenyls, Organochlorinated pesticides, Brominated diphenyl ethers

## Abstract

**Background:**

We are exposed to several chemicals such as persistent organic pollutants (POPs) in our everyday lives. Prior evidence has suggested that POPs may have adverse effects on reproductive function by disrupting hormone synthesis and metabolism. While there is age-related decline of fertility, the use of hormonal combined oral contraceptives (COCs) and its association to return of fertility remains controversial. The goal of this study is to investigate the association between exposure to POPs, both individually and as a mixture, and fecundability measured as time-to-pregnancy (TTP) according to pre-pregnancy use of COCs and age.

**Methods:**

Using the SELMA (Swedish Environmental Longitudinal Mother and Child, Allergy and Asthma) study, we have identified 818 pregnant women aged 18–43 years (mean 29 years) with data on how long they tried to get pregnant and what was their most recently used contraceptive method. These data were collected at enrollment to the study (median week 10 of pregnancy). Concentrations of 22 POPs and cotinine were analyzed in the blood samples collected at the same time as the questions on TTP and pre-pregnancy use of contraceptive. Analyses were done on the association between POPs exposure and TTP measured as continuous (months) and binary (infertile for those with TTP > 12 months). To study the chemicals individually, Cox regression and logistic regression were used to estimate fecundability ratios (FRs) and odds ratios (ORs), respectively. Weighted quantile sum (WQS) regression was used to investigate the chemicals as a mixture where chemicals of concern were identified above the 7.6% threshold of equal weights. To perform the subgroup analysis, we stratified the sample according to use of COCs as the most recent pre-pregnancy contraception method and age (< 29 years, and ≥ 29 years). The models were adjusted for parity, regularity of menses, maternal body mass index (BMI) and smoking status, and stratified as described above.

**Results:**

Prior to stratification, none of the POPs were associated with fecundability while increased exposure to HCB, PCB 74 and 118 had higher odds of infertility. Upon stratification, POP exposure was significantly associated with longer TTP in women aged ≥29 years who did not use COC. Specifically, PCBs 156, 180, 183, and 187 were associated with reduced fecundability while PCBs 99, 153, 156, 180, 183, and 187 had higher odds of infertility. As a mixture, we identified the chemicals of concern for a longer TTP include PCBs 118, 156, 183, and 187. Moreover, chemicals of concern identified with increased odds of infertility were PCB 74, 156, 183, 187, and transnonachlor.

**Conclusion:**

Serum concentrations of selected POPs, both as individual chemicals and as a mixture, were significantly associated with lower fecundability and increased odds of infertility in women aged 29 years and above not using COC as their most recent pre-pregnancy contraceptive. Our findings suggest that pre-pregnancy use of oral contraceptive and age may modify the link between POPs and fecundability. The differences of specific chemicals in the individual analysis and as a mixture support the need to study combination effects of chemicals when evaluating reproductive outcomes.

## Background

Persistent organic pollutants (POPs) such as organochlorine pesticides (OCPs), polychlorinated biphenyls (PCBs), and polybrominated diphenyl ethers (PBDEs) are organic substances that have long half-lives, bioaccumulate in the fatty tissues with increasing concentration towards the top of the food chain, and travel long distances in the atmosphere due to their semi-volatility [1]. They are widely used in agriculture, consumer products, and industrial products as well as unintentionally released as by-products from industrial processes and incineration. Exposure to these chemicals occurs through ingestion, inhalation, and absorption. We are exposed not only to a single chemical but to several chemicals simultaneously, resulting to mixtures that are associated with adverse outcomes [2–4]. Although many of these compounds have been strictly regulated in Europe including Sweden through the Stockholm Convention in 2001 [5], they still exist in the environment due to their high resistance to degradation. This has become a global health concern as studies of humans, wildlife populations, and epidemiological cohorts show associations between POP exposure and adverse effects on reproductive and endocrine functions [1, 6].

Since reproduction is regulated by hormones, infertility may occur when the endocrine system is disrupted. Infertility is the inability to achieve clinical pregnancy after 12 months of regular unprotected sexual intercourse [7]. By contrast, fertility is the actual production of a live offspring while fecundity is the ability to conceive given unprotected intercourse. Fecundability is the probability of becoming pregnant and is measured by time-to-pregnancy (TTP), which is the number of menstrual cycles or months without the use of contraceptives until a clinically detectable pregnancy is achieved [8, 9]. TTP has been used as a marker for fecundability of both parents to identify environmental exposures as potential hazards to human reproduction, encompassing series of biological events such as gametogenesis, fertilization, and implantation. Increased TTP may then indicate a problem in any or several of these stages [10].

There are several factors that affect female fertility. While biological factors such as age and BMI are inversely associated with fertility [11, 12], use of combined oral contraceptives (COCs), which is composed of both estrogen and progesterone, have contradicting studies about its association to return to fertility [13–15]. In addition, environmental risk factors such as exposure to POPs may also reduce fertility. Detection of environmental chemicals in human follicular fluid, serum, and seminal plasma led to the hypothesis of susceptibility of the reproductive function to disruption of biosynthesis and metabolism of hormones such as estrogen and progesterone [16, 17]. While POPs have been found to have effects on conception rates, menstrual cycles, and birth outcomes in animals such as rhesus monkeys and rodents [18, 19], previous human epidemiological studies have contradicting results. Some studies reported longer TTP for women exposed to POPs [20–22]. However, others did not find this association [23, 24]. These inconsistencies warrant further studies to explore the hazards of exposure to POPs and COC use.

### Aim of the study

The goal of this study is to investigate the association between exposure to POPs, both individually and as a mixture, during early pregnancy and fecundability measured as time-to-pregnancy (TTP) in regard to pre-pregnancy use of COCs and age.

## Methods

### Study population

This study utilized the Swedish Environmental Longitudinal, Mother and child, Asthma and allergy (SELMA) study, a pregnancy cohort with the primary aim to investigate the importance of early life exposure to environmental toxicants with focus on endocrine disrupting chemicals (EDCs) during the pregnancy and infancy period for health and development in the children [25]. Pregnant women were recruited from September 2007 to March 2010 during their first visit (median = 10 weeks age of gestation) at antenatal care centers in Värmland, Sweden. Out of 8394 women, 7119 were invited and 2582 (39%) consented to participate. Maternal blood was collected, and questionnaires were used to gather information regarding their medical history, lifestyles, and socioeconomic status. Questions on TTP were asked by midwife at enrollment. From the original cohort, only women with complete data on TTP, serum POP concentrations, age, parity, preventive method, regularity of menses, BMI, and lifestyle factors were included in this study (*n* = 818) (Supp Fig. 1).

### Time-to-pregnancy (TTP)

Fecundability or the probability of pregnancy in each cycle was measured through TTP, a sensitive and convenient marker used to study environmental exposures. Women were asked “How long have you been trying to get pregnant?” with answers in years and months. Additional questions on pre-pregnancy contraceptive use and reproductive history were also inquired. They were asked “What was the preventive method you used before you became pregnant?” and when they stopped using it. They were also asked if they have regular or irregular menses.

### Chemical analyses of POPs in serum

Concentrations of 22 POPs (nine OCPs namely pentachlorobenzene (PeCB), hexachlorobenzene (HCB), three isomers of hexachlorocyclohexane (α–HCH, β–HCH, γ–HCH), oxychlordane, transnonachlor, dichlorodiphenyltrichloroethane (p,p´-DDT), dichlorodiphenyldichloroethylene (p,p´-DDE), ten PCB congeners namely, PCB 74, 99, 118, 138, 153, 156, 170, 180, 183, and 187 as well as three PBDEs namely PBDE 47, 99, and 153) were analyzed in the blood samples collected during the first healthcare visit of current pregnancy, median 10 weeks age of gestation, as described previously [26]. Briefly, 200 ul of serum sample were enriched with 400 pg ^13^C-labelled internal standards of each compound. POPs were extracted with 2 ml dichloromethane-hexane (1:4). Extracts were cleaned with multilayer silica columns and the eluate was concentrated for gas chromatography – tandem mass spectrometry (GC-MS/MS) analysis (Agilent 7010 GC-MS/MS system, Wilmington, DE, USA). Control serum sample (NIST SRM 1958) and an in-house low-concentration control sample (1 to 9 dilution of SRM 1958 with new born calf serum) were included. Concentrations were reported as wet weight (pg/ml) and the limits of quantification (LOQ) ranged from 5 to 40 pg/ml. LOQ was defined as the concentration corresponding to ten times the standard deviation of the signal-to-noise ratio. Of the 22 POPs analyzed, 13 were detected in more than 70% of the included women.

### Analysis of cotinine in serum samples

Cotinine was measured in serum samples and used as a biomarker for tobacco smoke exposure. Isotopically labelled internal standards were added to 100 μl serum. Samples were digested with glucuronidase and proteins were precipitated using acetonitrile. Analyses were performed using triple quadrupole mass spectrometry (QTRAP 5500; AB Sciex, Foster City, USA) coupled to a liquid chromatography system (UFLCXR, Shimadzu Corporation, Kyoto, Japan) (LC-MS/MS). Subjects were categorized as non-smokers for cotinine concentrations below 0.2 ng/ml, passive smokers with cotinine concentrations 0.2–15 ng/ml and active smokers for cotinine above 15 ng/ml [27].

### Statistical analyses

POPs detected in less than 70% of the samples were not included in the analyses. Among those chemicals detected in more than 70% of the mothers, concentrations below the LOQ were replaced with LOQ/2. Characteristics of the cohort were presented as n (%), unless otherwise specified. Chi-square test, Wilcoxon rank-sum test and t-test test were used to analyze demographics and chemicals among groups. Correlation among chemicals were determined using Spearman’s rank correlation. To compare exposure patterns between Sweden and the United States, the geometric mean of serum concentrations of the 13 POPs were used to determine cumulative burden and its relative proportion in the SELMA cohort as well as in women aged 20–39 years in the National Health and Nutrition Examination Survey (NHANES) in 2007–2010 conducted by Centers for Disease Control and Prevention in the United States [28, 29].

Analyses were done on the association of POPs on the outcome variable TTP, both as continuous (measured in months) and binary (infertile for those with TTP > 12 months). Chemicals were analyzed both as continuous (log10 transformed) and quartiles. Discrete-time Cox regression models were used to estimate hazard ratios of fecundability (FR). The proportional hazards assumption was met based on the statistical tests on the scale Schoenfeld residuals. The FR represents the per-cycle probability of conception in subgroups with higher concentration POP exposure relative to a reference group with lower concentration POP exposure [30]. We censored the data when TTP exceeded 12 months. Sensitivity analyses were done with censored data at 10 and 14 months. An FR > 1 denotes higher fecundability, which means shorter TTP and FR < 1 denotes lower fecundability, which means longer TTP. Moreover, logistic regression was used to estimate odds ratio (OR) for infertility. An OR > 1 denotes higher odds for infertility and OR < 1 denotes lower odds for infertility. OR represents the odds that an outcome happens given a particular exposure compared to the odds of an outcome happening without that exposure [31]. To test the linear trend of the FR and OR, P-trend was calculated using the quartiles in a continuous form.

Aside from analyzing the individual compounds, a mixture approach was also performed. Due to the high correlation among chemicals, survival analysis was not used to explore mixture effects. Instead, weighted quantile sum (WQS) regression, a strategy for estimating empirical weights for a weighted sum of quantiled concentrations most associated with the health outcome (TTP and binary infertility), was performed [32]. Chemicals of concern were identified with a threshold of 7.6% (100/number of chemicals). Bootstrapping was set to 100. The dataset was split into a 35% training set and a 65% holdout validation set.

Because interaction tests between chemicals and covariates showed significant results for age and use of COCs, we stratified the sample based on these two factors, namely age (with the cohort mean 29 years old as cut off) and use of COC (yes, no) as the most recent pre-pregnancy contraception method. Quartiles were specific for each age group to ensure equal n in each quartile. All models were adjusted for well-established risk factors such as parity, education, regularity of menses (regular, irregular), maternal body mass index (BMI), and smoking status based on serum cotinine concentrations as described above. We also tested other covariates such as education (lower secondary, upper secondary/vocational studies, university/college), physical activity (< 1 h/week, 1 h/week, 1–2 h/week, ≥3 h/week), and alcohol intake (never, seldom, once every other week, at least once a week). The only paternal covariate that was tested was BMI. However, none of these other covariates modified the FR and OR by 10% or more. Hence, they were not added to the model.

These analyses were performed using IBM SPSS Statistics, version 22.0 (IBM Corp., Armonk, NY, USA) and the R programming language [33] through RStudio [34] with the packages gWQS [35], ggplot2 [36], ggpubr [37], and reshape2 [38].

## Results

Of the 818 women included in this study, the median TTP was 3 months with the interquartile range (25th to 75th percentile) between 1 and 6 months. Age was positively correlated with TTP (rho = 0.11, *p* = 0.002). Women who did not use COC had shorter TTP compared to women who used COC, both for those aged < 29 years (β = − 1, 95% CI = − 6.4, − 1) and aged ≥29 years (β = − 1, 95% CI = − 2, − 1) (Table 1). Seventy-nine women reported more than 12 months until pregnancy was achieved, resulting in a 9.7% censoring rate. Parity was higher among non-COC user women compared to COC user women. (Table 1).

**Table 1:** Cohort characteristics. Values are mean (SD), median (min-max) or n (%)

Characteristic		Total Cohort (*n* = 818)	< 29 years old		*p*-value*	≥ 29 years old		*p*-value*
			Non-COC user (*n* = 164)	COC user (*n* = 173)		Non-COC user (*n* = 280)	COC user (*n* = 201)	
Time to Pregnancy, months, median (min-max)		3 (0–84)	2 (0–48)	3 (0–36)	0.008	2 (0–84)	4 (0–84)	< 0.001
Age, yr, mean (SD)		29 (4.5)	25 (2.4)	25.5 (2.6)	0.03	32.9 (3.0)	31.7 (2.4)	< 0.001
Pre-pregnancy combined oral contraceptive use, n (%)		374 (45.7)	–	173		–	201	–
Parity, median (min-max)		0 (0–5)	0 (0–3)	0 (0–2)	< 0.001	1 (0–5)	0 (0–5)	< 0.001
Maternal BMI, kg/m^2^, median (min-max)		23.7 (16.9–45.4)	23.8 (17.3–40.1)	23.3 (17.2–39.3)	0.25	24.1 (16.9–45.4)	23.7 (18.3–39.8)	0.83
Paternal BMI, kg/m^2^, median (min-max)		25.6 (18.7–44.0)	25.4 (19.4–44.0)	25.5 (18.7–40.4)	0.98	25.7 (19.4–39.9)	25.8 (19.6–35.9)	0.96
Regularity of menses, n (%)	Regular	695 (85.0)	131 (79.9)	152 (87.9)	0.06	250 (89.3)	162 (80.6)	0.01
	Irregular	123 (15.0)	33 (20.1)	21 (12.1)		30 (10.7)	39 (19.4)	
Smoking, n (%)	Non smoker (Cotinine 0–0.2 ng/mL)	739 (90.3)	143 (87.2)	159 (91.9)	0.33	250 (89.3)	187 (93)	0.37
	Passive smoker (Cotinine 0.2–15 ng/mL)	38 (4.6)	9 (5.5)	7 (4)		15 (5.4)	7 (3.5)	
	Active smoker (Cotinine > 15 ng/mL)	41 (5.0)	12 (7.3)	7 (4)		15 (5.4)	7 (3.5)	
Alcohol, n (%)	Never	84 (10.3)	28 (17.1)	14 (8.1)	0.07	30 (10.7)	12 (6)	0.26
	Seldom	359 (43.9)	80 (48.8)	93 (53.8)		102 (36.4)	84 (41.8)	
	Once every other week	181 (22.1)	29 (17.7)	40 (23.1)		67 (23.9)	45 (22.4)	
	At least once a week	194 (23.7)	27 (16.5)	26 (15)		81 (28.9)	60 (29.9)	
Physical activity, n (%)	< 1 h/week	222 (27.1)	47 (28.7)	38 (22)	0.39	90 (32.1)	47 (23.4)	0.004
	1 h/week	133 (16.3)	27 (16.5)	28 (16.2)		54 (19.3)	24 (11.9)	
	1–2 h/week	220 (26.9)	41 (25)	42 (24.3)		74 (26.4)	63 (31.3)	
	at least 3 h/week	243 (29.7)	49 (29.9)	65 (37.6)		62 (22.1)	67 (33.3)	
Education, n (%)	Elementary	11 (1.3)	5 (3.0)	1 (0.6)	0.20	3 (1.1)	2 (1.0)	0.99
	High school/vocational	308 (37.7)	82 (50.0)	84 (48.6)		83 (29.6)	59 (29.4)	
	University/College	499 (61.0)	77 (47.0)	88 (50.9)		194 (69.3)	140 (69.7)	

Abbreviation: *BMI* body mass index, *COC* combined oral contraceptive

^*^Based on Wilcox, t.test or Chi-square test

**Fig. 1:**
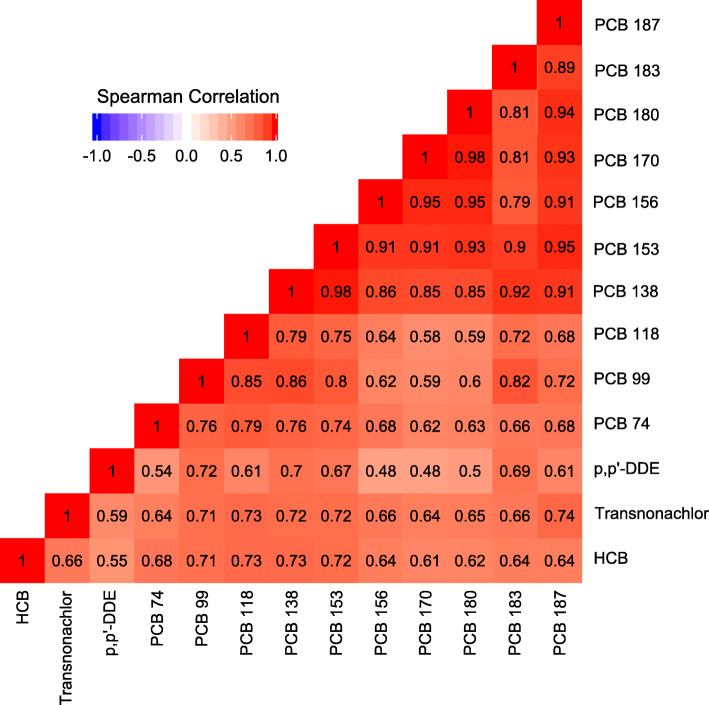
Correlation of POPs in maternal serum. Chemicals included are those detected in more than 70% of the samples. Data are presented as Spearman’s rank correlation rho ranging from −1.0 (dark blue) to 1.0 (dark red). All correlations are significant (*p* < 0.001). Number of observations can be found in Table 2. Abbreviations: HCB - hexachlorobenzene; p,p´-DDE - dichlorodiphenyldichloroethylene; PCB – polychlorinated biphenyl

**Fig. 2:**
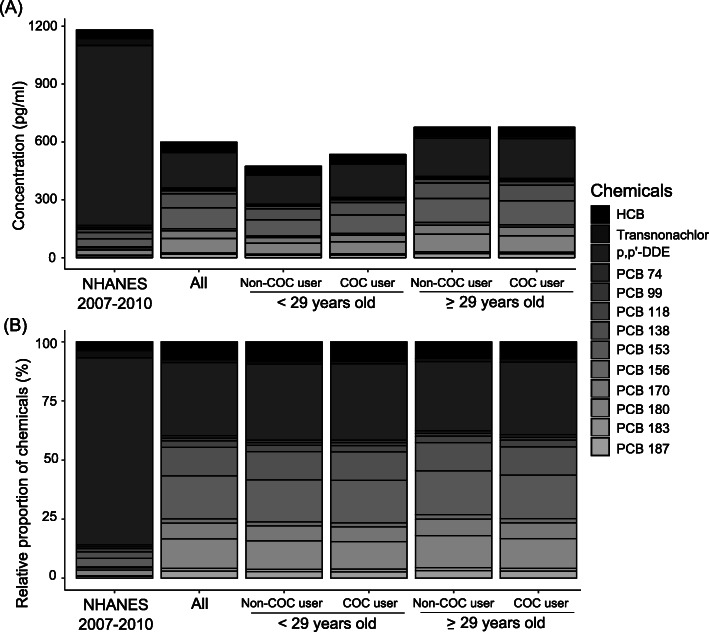
(**a**) Cumulative concentration and (**b**) relative proportion of POPs in NHANES 2007–2010 and SELMA cohort. Geometric means of serum concentrations of the 13 analyzed compounds were used to determine cumulative burden and relative proportion in women aged 20–39 years in NHANES conducted in 2007–2010 in the United States and Swedish SELMA cohort both unstratified and stratified. Abbreviations: POPs – persistent organic pollutants; COC – combined oral contraceptives; HCB - hexachlorobenzene; p,p´-DDE – dichlorodiphenyldichloroethylene; PCB – polychlorinated biphenyls; NHANES – National Health and Nutrition Examination Survey

Out of the 22 POPs studied, 13 chemicals, consisting of three OCPs and ten congeners of PCBs, were detected in more than 70% of the 818 women. Concentrations of the 13 chemicals were significantly positively correlated with each other (rho = 0.48–0.98, *p* < 0.001) (Fig. 1). The cumulative burden of serum POPs in the SELMA cohort varied from 500 to 650 pg/mL in the subgroups and was half of that of the women in the United States (Fig. 2). Similar exposure profiles were seen among all groups in the SELMA cohort, mainly consisting of p,p’-DDE (31%) followed by PCB 153 (18%), PCB 180 (13%), PCB 138 (12%), HCB (8%) and PCB 170 (7%). However, exposure profile in women in NHANES was mainly p,p’-DDE (80%) and HCB (4%) (Fig. 2) [28, 29]. Compared to exposure in women aged 20–39 years in the United States [28, 29], the SELMA cohort had lower concentrations of transnonachlor (*p* < 0.001), p,p’-DDE (*p* < 0.001), PCB 74 (*p* < 0.001), and 99 (*p* < 0.05) but higher concentrations of PCB 138 (*p* < 0.001), 153 (*p* < 0.001), 156 (*p* < 0.001), 170 (*p* < 0.001), 180 (*p* < 0.001), 183 (*p* < 0.001), and 187 (*p* < 0.001).

The serum concentrations of POPs between the groups were compared (Table 2). Among women < 29 years old, serum concentrations of HCB, transnonachlor, p,p’-DDE, PCBs 74, 99, 118, 138, 153, 156, 183, and PBDE 153 were lower in non-COC users compared to those of COC users. Among women ≥29 years old, there was no difference in POPs concentrations between non-COC and COC users (Table 2).

**Table 2:** Persistent organic pollutants (POPs) in maternal serum according to pre-pregnancy use of combined oral contraceptives (COCs) and age. Data are presented as GM (95% CI), pg/ml. Levels below LOQ replaced with LOQ/2

Chemical (*n* = 818)	LOQ (pg/mL)	n (%) > LOQ	Total Cohort (*n* = 818)	< 29 years old		*p*-value*	≥ 29 years old		*p*-value*
				Non-COC user (*n* = 164)	COC user (*n* = 173)		Non-COC user (*n* = 280)	COC user (*n* = 201)	
PeCB	10	0	–	–	–	–	–	–	–
HCB^a^	10	818 (100.0)	45.12 (44.15–46.12)	39.53 (37.65–41.51)	43.79 (42.02–45.64)	0.002	46.93 (45.16–48.76)	48.84 (46.84–50.93)	0.08
α-HCH	20	1 (0.1)	10.03 (9.97–10.09)	–	10.14 (9.86–10.43)	–	–	–	–
β-HCH	15	315 (38.5)	11.53 (11.05–12.03)	9.97 (9.19–10.82)	10.18 (9.23–11.22)	0.89	12.42 (11.54–13.38)	13.04 (12–14.17)	0.25
γ-HCH	20	0	–	–	–	–	–	–	–
Oxychlordane	25	3 (0.4)	12.55 (12.49–12.6)	12.62 (12.39–12.86)	12.5 (12.5–12.5)	0.31	12.56 (12.48–12.65)	12.5 (12.5–12.5)	0.23
Transnonachlor^a^	5	660 (80.7)	7.63 (7.28–7.99)	5.22 (4.69–5.81)	6.22 (5.68–6.81)	0.007	9.33 (8.69–10.01)	9.36 (8.56–10.24)	0.75
p,p’-DDT	15	49 (6.0)	8.15 (3.96–16.78)	8.04 (7.63–8.48)	7.98 (7.55–8.43)	0.52	8.27 (7.92–8.64)	8.22 (7.8–8.67)	0.80
p,p’-DDE^a^	40	816 (99.8)	184.68 (176.15–193.63)	152.19 (136.16–170.11)	172.73 (155.62–191.72)	0.03	198.45 (183.23–214.93)	207.26 (190.11–225.96)	0.33
PCB 74^a^	5	605 (74.0)	6.29 (6–6.59)	4.76 (4.28–5.3)	5.58 (5.06–6.15)	0.03	6.99 (6.5–7.52)	7.55 (6.89–8.27)	0.40
PCB 99^a^	5	665 (81.3)	7.27 (6.97–7.59)	5.59 (5.08–6.15)	6.6 (6.04–7.22)	0.01	8.09 (7.54–8.68)	8.45 (7.78–9.18)	0.58
PCB 118^a^	5	813 (99.4)	16.15 (15.61–16.72)	12.41 (11.54–13.35)	14.2 (13.23–15.24)	0.004	18.27 (17.28–19.32)	18.85 (17.68–20.1)	0.59
PCB 138^a^	5	818 (100.0)	72.01 (69.66–74.43)	56.67 (52.73–60.9)	64.81 (60.36–69.6)	0.02	80.98 (76.7–85.5)	81.4 (76.71–86.38)	0.93
PCB 153^a^	5	818 (100.0)	108.98 (105.4–112.68)	84.45 (78.45–90.92)	96.08 (89.38–103.29)	0.02	124.74 (118.26–131.59)	123.88 (116.85–131.33)	0.86
PCB 156^a^	5	739 (90.3)	10.97 (10.49–11.48)	7.91 (7.11–8.81)	9.19 (8.27–10.2)	0.03	13.32 (12.45–14.25)	12.74 (11.84–13.72)	0.49
PCB 170^a^	5	818 (100.0)	39.48 (38.04–40.97)	30.15 (27.74–32.77)	33.38 (30.74–36.26)	0.055	47.01 (44.39–49.79)	44.55 (41.82–47.46)	0.38
PCB 180^a^	5	818 (100.0)	75.3 (72.58–78.12)	56.73 (52.16–61.7)	62.28 (57.45–67.51)	0.08	91.3 (86.3–96.58)	85.41 (80.37–90.77)	0.20
PCB 183^a^	5	620 (75.8)	6.8 (6.5–7.12)	4.79 (4.35–5.28)	5.78 (5.27–6.35)	0.02	8.31 (7.72–8.95)	7.86 (7.21–8.56)	0.31
PCB 187^a^	5	802 (98.0)	17.76 (17.06–18.5)	13.07 (11.88–14.37)	14.53 (13.24–15.96)	0.07	21.97 (20.71–23.31)	20.17 (18.88–21.54)	0.06
PBDE 47	15	40 (5.0)	8.01 (7.84–8.19)	7.8 (7.55–8.05)	8.26 (7.78–8.77)	0.24	8 (7.69–8.32)	7.99 (7.66–8.33)	0.99
PBDE 99	15	10 (1.2)	7.62 (7.54–7.69)	7.5 (7.5–7.5)	7.72 (7.5–7.96)	0.05	7.57 (7.47–7.68)	7.69 (7.5–7.88)	0.21
PBDE 153	15	17 (2.1)	7.76 (4.64–12.98)	7.54 (7.47–7.61)	7.89 (7.59–8.2)	0.04	7.91 (7.59–8.24)	7.62 (7.39–7.86)	0.06

^a^Chemicals detected in more than 70% of the samples above the LOQ were included in further analyses

*Based on Wilcoxon rank sum test

Abbreviations: *CI* confidence interval, *GM* geometric mean, *LOQ* limit of quantification, *PeCB* pentachlorobenzene, *HCB* hexachlorobenzene, *HCH *hexachlorocyclohexane, *p,p´-DDT* dichlorodiphenyltrichloroethane, *p,p´-DDE* dichlorodiphenyldichloroethylene, *PCB* polychlorinated biphenyls, *PBDE* polybrominated diphenyl ether

**Fig. 3:**
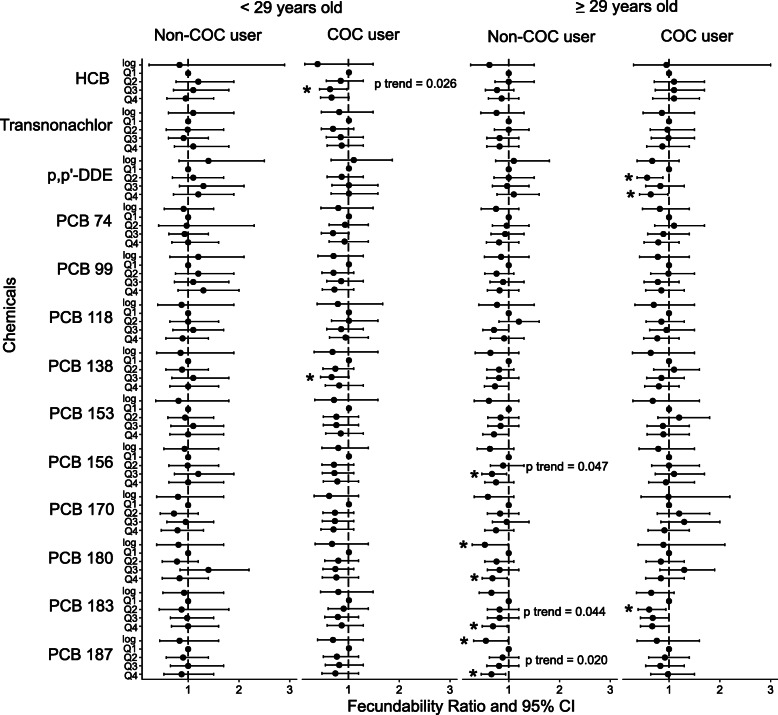
Associations of POPs exposure with TTP using Cox regression. Dot shows fecundability ratio (FR) with 95% confidence interval error bars of chemicals as continuous (log) and quartiles (Q1 as reference). FR > 1 denotes higher fecundability and shorter TTP while FR < 1 denotes lower fecundability and longer TTP. FR indicated with * for p value < 0.05. P trend shown for significant linear trend of FR. Abbreviations: COC – combined oral contraceptives; HCB - hexachlorobenzene; p,p´-DDE – dichlorodiphenyldichloroethylene; PCB – polychlorinated biphenyls; Q1 – first quartile, Q2 – second quartile, Q3 – third quartile, Q4 – fourth quartile

Without age and COC stratification, none of the chemicals (as continuous or quartiles) had significant FR after adjustment with age, pre-pregnancy use of COCs, parity, maternal BMI and smoking status (Suppl Table 1). However, upon stratification, significant associations were found. Among women aged < 29 years who used COC, those exposed within the third quartiles of HCB and PCB 138 had 37% reduced fecundability (i.e. longer TTP) compared to those exposed to their respective first quartiles (FR 0.63; 95% CI 0.4–0.99 and FR 0.63; 95% CI 0.4–0.98, respectively) (Fig. 3). Among women aged ≥29 years who did not use COC, those exposed in the third quartile of PCB 156 (FR 0.67; 95% CI 0.46–0.95), and fourth quartiles of PCB 180 (FR 0.69; 95% CI 0.48–0.98), 183 (FR 0.69; 95% CI 0.49–0.98) and 187 (FR 0.66; 95% CI 0.46–0.95) had longer TTP compared their respective first quartiles (Fig. 3). There was a 47% (FR 0.53; 95% CI 0.28–0.99) and 45% (FR 0.55; 95% CI 0.31–0.98) decrease in fecundability for every 10-fold increase of PCB 180 and 187, respectively (Fig. 3). On the other hand, older women who used COCs had a reduction in fecundability for those in the second and fourth quartiles of p,p’-DDE (FR 0.56; 95% CI 0.36–0.87 and FR 0.65; 95% CI 0.42–0.99, respectively), and second quartile of PCB 183 (FR 0.60; 95% CI 0.39–0.93) compared to their quartiles (Fig. 3). Sensitivity analyses with censored data at 10 and 14 months showed similar results (Supp Figs. 2 and 3).

**Fig. 4:**
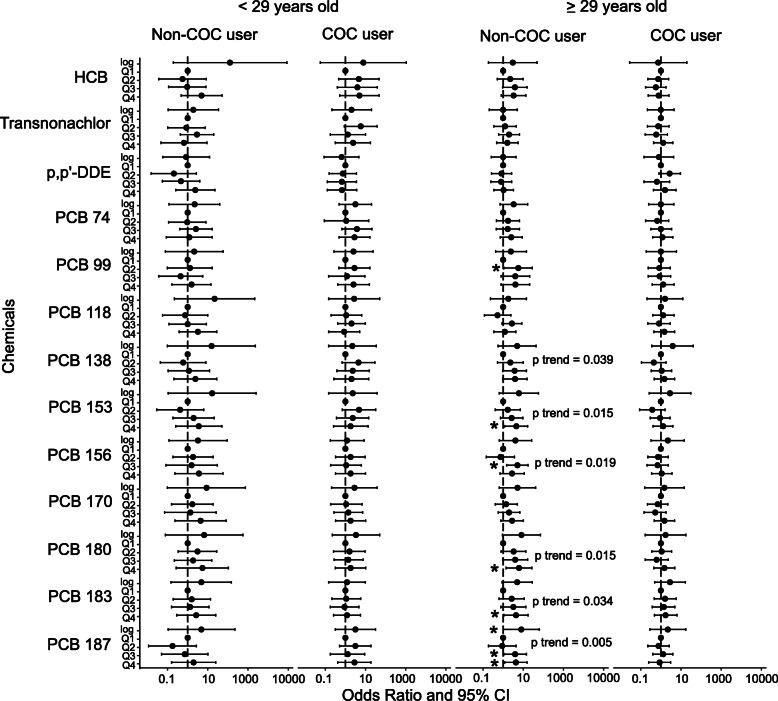
Associations of POPs exposure with infertility using logistic regression. Dot shows odds ratio (OR) with 95% confidence interval (CI) error bars of chemicals as continuous (log) and quartiles (Q1 as reference). OR > 1 denotes higher odds for infertility while OR < 1 denotes lower odds for infertility. OR indicated with * for *p* value < 0.05. P trend shown for significant linear trend of OR. Abbreviations: COC – combined oral contraceptives; HCB - hexachlorobenzene; p,p´-DDE – dichlorodiphenyldichloroethylene; PCB – polychlorinated biphenyls; Q1 – first quartile, Q2 – second quartile, Q3 – third quartile, Q4 – fourth quartile

Prior to age and COC stratification, every 10-fold increase in exposure to PCB 74 and 118 had a 3-fold (OR 3; 95% CI 1.33, 6.79) and 4-fold (OR 4.06; 95% CI 1.28, 12.88) odds to be infertile, respectively (Suppl Table 1). The third quartile of HCB had 125% (OR 2.25; 95% CI 1.06, 4.78) higher odds to be infertile compared to the first quartile (Suppl Table 1). After stratification, every 10-fold increase in exposure to PCB 187 had an 8-fold higher odds for infertility in women aged ≥29 years old who did not use COC (OR 7.99; 95% CI 1.02, 62.83) (Fig. 4). There were also higher odds for infertility for those in the second quartile of PCB 99 (OR 5.62; 95% CI 1.15, 27.5), fourth quartile of PCB 153 (OR 4.53; 95% CI 1.23, 16.64), third quartile of PCB 156 (OR 5.04; 95% CI 1.47, 17.22), fourth quartile of PCB 180 (OR 6.08; 95% CI 1.42, 26.07), fourth quartile of PCB 183 (OR 4.38; 95% CI 1.11, 17.21), and third and fourth quartiles of PCB 187 (OR 3.87; 95% CI 1.11, 13.5 and OR 4.26; 95% CI 1.17, 15.48) (Fig. 4). However, none of the chemicals was significantly associated to infertility in the three other groups of women (Fig. 4).

**Fig. 5:**
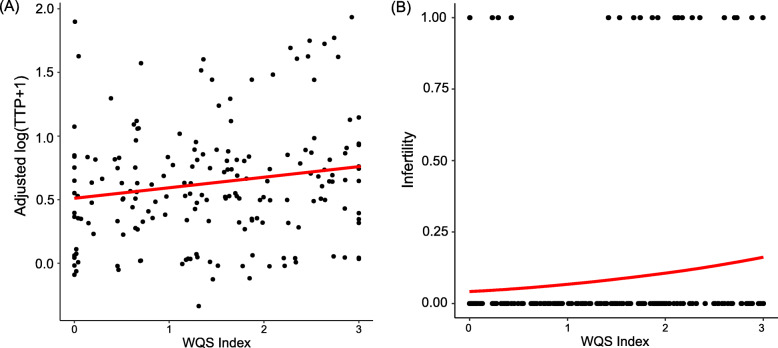
Association between POP mixture and fertility outcomes in women ≥29 years old not using COCs. Graph shows shape and direction of association between weighted quantile sum (WQS) index and (**a**) time-to-pregnancy and (**b**) infertility. Logistic regression fit was for women with parity = 1, non-smoker, regular menses, and BMI = 24 kg/m^2^

Because the chemicals were highly correlated (Fig. 1), WQS regression was performed to study the exposure to POPs as a mixture. The WQS-index was not significantly associated with TTP nor infertility prior to stratification. However, after stratification, there was a 9% increase of the log transformed TTP for every one quartile increase of the WQS-index in women aged ≥29 years old not using COCs (β = 0.09; SE = 0.04; *p* < 0.05) (Fig. 5 and Table 3). Identified chemicals of concern (i.e. weights above 7.6% cutoff) were PCB 183 (35.6%), PCB 156 (23.4%), PCB 187 (14.7%) and PCB118 (10.9%). There was also a 79% increase in odds for infertility (OR 1.79; 95% CI 1.03, 3.11) (Fig. 5 and Table 3). Chemicals of concern were PCB 187 (42.2%), PCB 156 (22.1%), PCB 74 (9.8%), PCB 183 (9.2%), and transnonachlor (8.0%). None of the WQS-indexes were significantly associated with TTP nor infertility in the three other groups of women.

**Table 3:** Weighted quantile sum index stratified according to age and use of combined oral contraceptives (COCs). Data presented are estimate (SE) and OR (95% CI)

Group		Outcome: TTP^a^ Estimate (SE)^b^	Chemicals of concern^c^	Outcome: Infertility OR (95% CI) ^b^	Chemicals of concern^c^
< 29 years old	Non-COC user	−0.02(0.05)	–	0.97 (0.22–4.26)	–
	COC user	0.03 (0.03)	–	0.92 (0.35–2.41)	–
≥ 29 years old	Non-COC user	0.09 (0.03)*	PCB 183, 156, 187, 118	1.79 (1.03–3.11)*	PCB 187, 156, 74, 183, transnonachlor
	COC user	0.07 (0.04)	–	1.27 (0.07–2.3)	–

^a^log transformed + 1

^b^Adjusted with parity, regularity of menses, maternal BMI, and smoking

^c^Chemicals with weight greater than 7.6%

* *p* < 0.05

Abbreviations: *CI* confidence interval, *COCs* combined oral contraceptives, *PCB* polychlorobiphenyl, *OR* odds ratio, *SE* standard error, *TTP* time-to-pregnancy

## Discussion

The main finding of our study showed that serum concentrations of certain POPs were significantly associated with lower fecundability and higher odds of infertility in women aged ≥29 years who did not use COC as their most recent pre-pregnancy contraceptive even though none of the POPs were associated with fecundability prior to stratification. Our findings suggest that pre-pregnancy use of combined oral contraceptives and age may modify the link between POPs and fecundability. To our knowledge, this is the first study to investigate the chemicals both individually and as a mixture as well as focus on POPs exposure and the effect modification by pre-pregnancy COC use and age in relation to fecundability.

In the individual chemical stratified analyses, higher concentrations of PCBs 153, 180, 183 and 187 were associated either with lower fecundability and/or higher odds for infertility in at least one stratum. However, this monotonic dose response was not observed with HCB, p,p’-DDE, PCBs 99, 138, 156, and 183, where only either the second or third quartiles were significant, but not the fourth quartile. Nonetheless, these chemicals were still associated with reduced fecundability, albeit challenging conventional toxicological dogmas such as monotonicity. There was a positive correlation among the chemicals analyzed, where higher exposure to one POP was linked to increased exposure to other POPs. This demonstrates the need for taking mixture exposures into account since we are exposed to several compounds at the same time. In the current study, we used the WQS regression in determining the chemicals of concern in mixtures that were associated with longer TTP and infertility, which was significant among women aged ≥29 years not using COCs. In this stratum, while the chemicals in the individual analyses were in part similar to those chemicals of concern in the mixture (i.e. PCB 156, 183 and 187 for both TTPand infertility), there were still chemicals that were significant only for the individual analyses (i.e. PCB 180 for TTP; PCB 99, 153, and 180 for infertility) but not for the mixtures and vice versa (i.e. PCB 118 for TTP; PCB 74 and transnonachlor for infertility). Hence, it is important to study chemicals as a mixture because possible antagonistic, synergistic or additive properties between chemicals may be overlooked when chemicals are assessed individually, which may lead to underestimation of hazards and risks [39]. As of yet, there is no single overarching systematic, comprehensive, and integrated approach in understanding mixtures but there is continuous effort globally to develop strategies in studying mixtures. To quantify the effects of mixtures, several statistical approaches have been proposed from simple linear regression to advanced machine learning techniques but there is no one particular method that outperforms the other [40]. This study supports the need to design and optimize approaches in addressing mixtures in epidemiological studies.

Comparison of our results to previous findings is challenging. We have found that most previous studies only utilized pre-pregnancy use of COCs to adjust their model but did not examine it further through stratification. Nonetheless, these studies have shown associations of POPs to longer TTP [23, 24] while other studies failed to replicate these results [20, 22]. Similar to our results, it has also been shown that PCB mixtures were associated with longer TTP [41]. In our study, stratification allowed studying the association of the chemicals in four homogenous groups taking into account two important factors: age and COC use. While this approach prevented direct comparison between groups, we were able to draw specific inferences on associations of chemicals on each stratum.

Previous studies show inconclusive evidence on return to fertility after cessation of COCs. While it was seen that TTP in former COC users were comparable with other contraceptive methods [13], another study showed reduction in TTP after COC use [14]. On the other hand, duration of use may play a role in this association as it was also shown that short-term use of COC may cause delay in return-to-fertility or longer TTP while long-term use of COC has no deleterious effects on fecundability [15]. However, other studies have shown that external factors such as contraceptive use and lifestyle were not as important predictors of TTP as biological factors such as age of conception and menstrual cycle length [42]. Nonetheless, our findings show that both COC use and age play a role on TTP and its relationship with POPs.

The proportions of chemicals differed between SELMA and NHANES, showing that exposure patterns may vary between countries. While both cohorts were of similar reproductive age and time period, other factors such as occupation, race and lifestyle factors may contribute to these differences. This further emphasizes the need to focus further studies on mixture approach in analyzing chemicals exposure. Importantly, POPs are not the only chemicals women of reproductive age are exposed to. Recent biomonitoring studies consistently find more than 300 environmental chemicals or their metabolites in the serum of nearly all analyzed individuals [43]. It is of high priority to study how extensive mixtures of chemicals collectively modulate reproductive functions in women.

### Possible biological mechanism

One of the molecular mechanisms that possibly could explain our results is the well-known impact of COCs on adenosine triphosphate (ATP)-binding cassette (ABC) transporters [44]. ABC transporters are highly conserved transmembrane proteins that are responsible for the efflux of substances towards the extracellular space. With this efflux, substances are eliminated and prevented from accumulating inside the cell [45]. While it aids in many physiological processes such as steroid production and immunological processes, it also provides support by efflux of environmental toxins out of the cells [46]. A number of ABC proteins, such as P-glycoprotein encoded by the *ABCB1* gene, were shown to be upregulated by estrogen and progesterone (i.e. hormones that are present in COCs) [44, 47]. With the increased production of the ABC transporters, environmental chemicals such as POPs could possibly be eliminated from the cell [46], suggesting to less toxic effects on reproductive functions. It has also been shown that there is an age-related downregulation of ABC transporters [48], which possibly explains observing the association mostly in women older than 29 years old. Further studies are warranted to confirm this hypothesis.

### Limitations of the study

Our study has limitations. The chemical concentrations were not lipid adjusted. In addition, our study focused on combined oral contraceptives, excluding other hormonal contraception methods such as progesterone only pills and intrauterine device with hormones. There was neither data on the duration and type of the COC use prior to discontinuation. Moreover, while we recognize that paternal factors may also affect TTP, the only paternal data in the cohort was BMI, which was eventually not included in the model because it did not modify the FR more than 10%. Unplanned pregnancies were also not available in the cohort. Lack of these data may lead to overestimation of findings, hence, further studies are needed to confirm these results. Because this study utilized a pregnancy cohort, the least fertile couples were less represented. In addition, different statistical analyses were performed when studying the chemicals as individual compounds and as a mixture. While Cox proportional hazard was not a problem for possible presence of non-monotonic dose response, it cannot handle highly correlated predictors and cannot be used for mixture analysis. On the other hand, WQS was appropriate for handling intracollinearity but limited to monotonic dose response. Hence, comparison of results between the analyses must be taken with caution.

## Conclusions

Although the studied 13 chemicals were banned several decades ago, many of them were still detected in majority of the serum samples collected in 818 pregnant women during the period 2007–2010 living in Sweden. Even though none of the POPs were associated with fecundability prior to stratification, our results indicate that higher POP exposure, both as individual chemicals and as a mixture, may be associated with lower fecundability and increased infertility in women ≥29 years old not using COC as their most recent pre-pregnancy contraception method. This is important as more and more women postpone childbearing, which could make the chemical effects more profound. This observation could also help explain some of the contradictory findings in the literature. Use of COCs may modify the relationship between POPs and TTP. This study also supports studying mixtures in epidemiological studies to further understand combination effects of chemicals.

## Supplementary information

**Additional file 1: Table S1.** Estimated fecundability ratio (FR) for time-to-pregnancy (TTP) and odds ratio (OR) for infertility prior to stratification. Data are presented as FR (95% CI) or OR (95% CI). Supp **Figure S1.** Flowchart of recruitment process and analyses in the SELMA study. Abbreviations: COC – combined oral contraceptives; FR – fecundability ratio; OR – odds ratio; POPs – persistent organic pollutants; TTP – time-to-pregnancy; WQS – weighted quantile sum. Supp **Figure S2.** Sensitivity analyses with censoring at 10 months. Dot shows fecundability ratio (FR) with 95% confidence interval error bars of chemicals as continuous (log) and quartiles (Q1 as reference). FR > 1 denotes higher fecundability and shorter TTP while FR < 1 denotes lower fecundability and longer TTP. FR indicated with * for *p* value < 0.05. P trend shown for significant linear trend of FR. Abbreviations: COC – combined oral contraceptives; HCB - hexachlorobenzene; p,p´-DDE – dichlorodiphenyldichloroethylene; PCB – polychlorinated biphenyls; Q1 – first quartile, Q2 – second quartile, Q3 – third quartile, Q4 – fourth quartile. Supp **Figure S3.** Sensitivity analyses with censoring at 14 months. Dot shows fecundability ratio (FR) with 95% confidence interval error bars of chemicals as continuous (log) and quartiles (Q1 as reference). FR > 1 denotes higher fecundability and shorter TTP while FR < 1 denotes lower fecundability and longer TTP. FR indicated with * for p value < 0.05. P trend shown for significant linear trend of FR. Abbreviations: COC – combined oral contraceptives; HCB - hexachlorobenzene; p,p´-DDE – dichlorodiphenyldichloroethylene; PCB – polychlorinated biphenyls; Q1 – first quartile, Q2 – second quartile, Q3 – third quartile, Q4 – fourth quartile.

## Data Availability

All data generated or analysed during this study are included in this published article and its supplementary information files.

## Abbreviations

ABC: ATP-binding cassette
ATP: Adenosine triphosphate
BMI: Body mass index
CI: Confidence interval
COCs: Combined oral contraceptives
FR: Fecundability ratio
GM: Geometric mean
HCB: Hexachlorobenzene
HCH: Hexachlorocyclohexane
GC-MS/MS: Gas chromatography – tandem mass spectrometry
LC-MS/MS: Liquid chromatography/ triple quadrupole mass spectrometry
LOQ: Limit of quantification
NHANES: National Health and Nutrition Examination Survey
OCP: Organochlorine pesticide
OR: Odds ratio
PBDE: Polybrominated diphenyl ether
PCB: Polychlorinated biphenyl
PeCB: Pentachlorobenzene
POPs: Persistent organic pollutants
p,p´-DDT: Dichlorodiphenyltrichloroethane
p,p´-DDE: Dichlorodiphenyldichloroethylene
SELMA: Swedish Environmental Longitudinal, Mother and child, Asthma and allergy
SD: Standard deviation
SE: Standard error
TTP: Time-to-pregnancy
