# Supplementary material for: Persistent organic pollutants, pre-pregnancy use of combined oral contraceptives, age, and time-to-pregnancy in the SELMA cohort
Source: Environ Health. 2020 Jun 15;19:67. doi: 10.1186/s12940-020-00608-8 (PMC7294652; doi:10.1186/s12940-020-00608-8)
Supplement: Supplementary file 1 — Additional file 1: Table S1. Estimated fecundability ratio (FR) for time-to-pregnancy (TTP) and odds ratio (OR) for infertility prior to stratification. Data are presented as FR (95% CI) or OR (95% CI). Supp Figure S1. Flowchart of recruitment process and analyses in the SELMA study. Abbreviations: COC – combined oral contraceptives; FR – fecundability ratio; OR – odds ratio; POPs – persistent organic pollutants; TTP – time-to-pregnancy; WQS – weighted quantile sum. Supp Figure S2. Sensitivity analyses with censoring at 10 months. Dot shows fecundability ratio (FR) with 95% confidence interval error bars of chemicals as continuous (log) and quartiles (Q1 as reference). FR > 1 denotes higher fecundability and shorter TTP while FR < 1 denotes lower fecundability and longer TTP. FR indicated with * for p value < 0.05. P trend shown for significant linear trend of FR. Abbreviations: COC – combined oral contraceptives; HCB - hexachlorobenzene; p,p´-DDE – dichlorodiphenyldichloroethylene; PCB – polychlorinated biphenyls; Q1 – first quartile, Q2 – second quartile, Q3 – third quartile, Q4 – fourth quartile. Supp Figure S3. Sensitivity analyses with censoring at 14 months. Dot shows fecundability ratio (FR) with 95% confidence interval error bars of chemicals as continuous (log) and quartiles (Q1 as reference). FR > 1 denotes higher fecundability and shorter TTP while FR < 1 denotes lower fecundability and longer TTP. FR indicated with * for p value < 0.05. P trend shown for significant linear trend of FR. Abbreviations: COC – combined oral contraceptives; HCB - hexachlorobenzene; p,p´-DDE – dichlorodiphenyldichloroethylene; PCB – polychlorinated biphenyls; Q1 – first quartile, Q2 – second quartile, Q3 – third quartile, Q4 – fourth quartile. [file 12940_2020_608_MOESM1_ESM.docx]

# Persistent organic pollutants, pre-pregnancy use of combined oral contraceptive, age and time-to-pregnancy in the SELMA cohort

**Richelle D. Björvang**, Chris Gennings, Ping-I Lin, Ghada Hussein, Hannu Kiviranta, Panu Rantakokko, Päivi Ruokojärvi, Pauliina Damdimopoulou, Carl-Gustaf Bornehag

**Supplementary material**

**Supplementary Table 1. Estimated fecundability ratio (FR) for time-to-pregnancy (TTP) and odds ratio (OR) for infertility prior to stratification. Page 2**

**Supplementary Figure 1. Flowchart of recruitment process and analyses in the SELMA study**

**Page 4**

**Supplementary Figure 2. Sensitivity analyses with censoring at 10 months Page 5**

**Supplementary Figure 3. Sensitivity analyses with censoring at 14 months Page 6**

**Supplementary Table 1.** Estimated fecundability ratio (FR) for time-to-pregnancy (TTP) and odds ratio (OR) for infertility prior to stratification. Data are presented as FR (95% CI) or OR (95% CI).

| **Chemical** | | **Unadjusted FR^a^** | **Adjusted FR^a.c^** | **Infertility^b^** | | |
| --- | --- | --- | --- | --- | --- | --- |
|  |  |  |  | **Unadjusted OR** | **Adjusted OR^c^** |  |
| HCB | Continuous^d^ | 0.57 (0.34-0.98)* | 0.95 (0.53-1.7) | 5.08 (0.98-26.43) | 2.11 (0.3-15.07) |  |
|  | Q1 (<37.04) | 1 | 1 | 1 | 1 |  |
|  | Q2 (37.04-44.94) | 0.94 (0.77-1.15) | 0.97 (0.79-1.19) | 1.35 (0.62-2.94) | 1.38 (0.61-3.12) |  |
|  | Q3 (44.95-54.85) | 0.72 (0.59-0.89)** | 0.81 (0.66-1.01) | 2.64 (1.31-5.33)** | 2.25 (1.06-4.78)* |  |
|  | Q4 (>54.86) | 0.85 (0.69-1.04) | 1.02 (0.81-1.27) | 1.93 (0.93-4.02) | 1.29 (0.57-2.91) |  |
| Trans  nonachlor | Continuous^d^ | 0.8 (0.63-1.03) | 1.15 (0.85-1.56) | 1.84 (0.83-4.07) | 0.69 (0.26-1.87) |  |
|  | Q1 (<5.5) | 1 | 1 | 1 | 1 |  |
|  | Q2 (5.5-8.17) | 0.88 (0.72-1.08) | 0.97 (0.78-1.19) | 1.55 (0.76-3.14) | 1.19 (0.56-2.54) |  |
|  | Q3 (8.18-11.91) | 0.87 (0.71-1.06) | 1.03 (0.82-1.28) | 1.47 (0.72-2.99) | 0.97 (0.44-2.11) |  |
|  | Q4 (>11.92) | 0.86 (0.7-1.05) | 1.07 (0.84-1.37) | 1.81 (0.91-3.61) | 0.99 (0.44-2.23) |  |
| p.p'-DDE | Continuous^d^ | 0.98 (0.76-1.26) | 1.23 (0.95-1.6) | 1.13 (0.53-2.41) | 0.59 (0.24-1.49) |  |
|  | Q1 (<117.63) | 1 | 1 | 1 | 1 |  |
|  | Q2 (117.63-173.39) | 0.93 (0.76-1.14) | 1.07 (0.87-1.32) | 0.87 (0.43-1.77) | 0.65 (0.31-1.37) |  |
|  | Q3 (173.4-263.18) | 0.87 (0.71-1.07) | 1.03 (0.83-1.28) | 1.31 (0.68-2.5) | 0.9 (0.43-1.84) |  |
|  | Q4 (>263.19) | 0.93 (0.76-1.14) | 1.15 (0.92-1.43) | 1.25 (0.65-2.41) | 0.75 (0.35-1.6) |  |
| PCB 74 | Continuous^d^ | 0.69 (0.54-0.89)** | 0.94 (0.72-1.23) | 3.05 (1.42-6.51)* | 3 (1.33-6.79)** |  |
|  | Q1 (<2.5) | 1 | 1 | 1 | 1 |  |
|  | Q2 (2.5-6.68) | 0.9 (0.74-1.1) | 1.03 (0.83-1.26) | 1.29 (0.58-2.86) | 0.99 (0.43-2.29) |  |
|  | Q3 (6.69-9.55) | 0.79 (0.65-0.97)* | 0.95 (0.77-1.18) | 2.65 (1.31-5.37)** | 1.93 (0.9-4.15) |  |
|  | Q4 (>9.56) | 0.77 (0.63-0.94)* | 0.99 (0.79-1.25) | 2.34 (1.14-4.79)* | 1.38 (0.61-3.12) |  |
| PCB 99 | Continuous^d^ | 0.76 (0.58-0.99) | 1.05 (0.78-1.42) | 2.88 (1.16-7.15)* | 1.38 (0.48-3.94) |  |
|  | Q1 (<5.68) | 1 | 1 | 1 | 1 |  |
|  | Q2 (5.68-7.88) | 0.96 (0.79-1.18) | 1.01 (0.82-1.24) | 0.85 (0.4-1.84) | 0.72 (0.32-1.62) |  |
|  | Q3 (7.89-10.96) | 0.85 (0.69-1.04) | 1.01 (0.81-1.26) | 1.59 (0.81-3.15) | 1.11 (0.53-2.33) |  |
|  | Q4 (>10.97) | 0.8 (0.65-0.98)* | 1 (0.79-1.25) | 2 (1.04-3.88)* | 1.21 (0.57-2.58) |  |
| PCB 118 | Continuous^d^ | 0.66 (0.47-0.91)* | 0.99 (0.68-1.45) | 4.54 (1.53-13.49)** | 4.06 (1.28-12.88)* |  |
|  | Q1 (<11.63) | 1 | 1 | 1 | 1 |  |
|  | Q2 (11.63-15.97) | 0.99 (0.81-1.21) | 1.1 (0.89-1.35) | 1.16 (0.54-2.5) | 0.91 (0.41-2.04) |  |
|  | Q3 (15.98-22.56) | 0.81 (0.66-0.99)* | 0.98 (0.79-1.22) | 2.12 (1.06-4.26)* | 1.33 (0.63-2.82) |  |
|  | Q4 (>22.57) | 0.82 (0.67-1) | 1.04 (0.83-1.31) | 2.06 (1.02-4.16)* | 1.08 (0.49-2.39) |  |
| PCB 138 | Continuous^d^ | 0.64 (0.45-0.91)* | 0.9 (0.6-1.36) | 4.74 (1.52-14.84)** | 2.74 (0.69-10.83) |  |
|  | Q1 (<53.75) | 1 | 1 | 1 | 1 |  |
|  | Q2 (53.75-71.98) | 0.93 (0.77-1.14) | 0.98 (0.8-1.21) | 0.91 (0.42-2) | 0.81 (0.36-1.86) |  |
|  | Q3 (71.99-98.76) | 0.87 (0.71-1.06) | 1 (0.8-1.24) | 1.64 (0.81-3.3) | 1.43 (0.66-3.07) |  |
|  | Q4 (>98.77) | 0.75 (0.61-0.92)** | 0.9 (0.71-1.14) | 2.34 (1.2-4.56)* | 1.75 (0.79-3.88) |  |
| PCB 153 | Continuous^d^ | 0.67 (0.47-0.94)* | 0.93 (0.61-1.41) | 3.85 (1.24-11.94)* | 2.5 (0.62-10.16) |  |
|  | Q1 (<81.59) | 1 | 1 | 1 | 1 |  |
|  | Q2 (81.59-110.73) | 0.86 (0.71-1.06) | 0.93 (0.76-1.15) | 1.07 (0.5-2.28) | 0.89 (0.4-1.99) |  |
|  | Q3 (110.74-149.8) | 0.87 (0.71-1.06) | 0.99 (0.8-1.24) | 1.63 (0.81-3.29) | 1.39 (0.64-3.03) |  |
|  | Q4 (>149.81) | 0.76 (0.62-0.93)** | 0.9 (0.71-1.14) | 2.16 (1.1-4.23)* | 1.73 (0.76-3.95) |  |
| PCB 156 | Continuous^d^ | 0.79 (0.61-1.02) | 0.99 (0.73-1.35) | 1.96 (0.83-4.63) | 1.4 (0.49-4.03) |  |
|  | Q1 (<8.09) | 1 | 1 | 1 | 1 |  |
|  | Q2 (8.09-11.97) | 0.9 (0.73-1.1) | 0.98 (0.8-1.22) | 0.99 (0.48-2.05) | 0.89 (0.41-1.94) |  |
|  | Q3 (11.98-16.88) | 0.89 (0.73-1.09) | 1.04 (0.83-1.29) | 1.41 (0.72-2.78) | 1.07 (0.5-2.27) |  |
|  | Q4 (>16.89) | 0.83 (0.68-1.02) | 0.97 (0.76-1.22) | 1.64 (0.85-3.18) | 1.39 (0.62-3.1) |  |
| PCB170 | Continuous^d^ | 0.77 (0.56-1.05) | 0.96 (0.66-1.4) | 2.2 (0.8-6.08) | 1.68 (0.47-5.97) |  |
|  | Q1 (<28.68) | 1 | 1 | 1 | 1 |  |
|  | Q2 (28.68-41.11) | 0.91 (0.74-1.11) | 0.93 (0.75-1.15) | 1.15 (0.54-2.46) | 1.42 (0.66-3.06) |  |
|  | Q3 (41.12-56.17) | 0.92 (0.75-1.12) | 1.02 (0.82-1.26) | 0.95 (0.43-2.13) | 1.15 (0.53-2.49) |  |
|  | Q4 (>56.18) | 0.85 (0.69-1.04) | 0.95 (0.75-1.2) | 1.7 (0.73-3.99) | 1.59 (0.7-3.64) |  |
| PCB 180 | Continuous^d^ | 0.76 (0.56-1.04) | 0.95 (0.64-1.42) | 2.38 (0.86-6.64) | 1.99 (0.52-7.62) |  |
|  | Q1 (<54.17) | 1 | 1 | 1 | 1 |  |
|  | Q2 (54.17-77.69) | 0.99 (0.81-1.22) | 1.04 (0.84-1.29) | 1.06 (0.53-2.12) | 1.15 (0.54-2.46) |  |
|  | Q3 (77.7-106.22) | 0.96 (0.78-1.17) | 1.04 (0.83-1.3) | 0.99 (0.49-2.01) | 0.95 (0.43-2.13) |  |
|  | Q4 (>106.23) | 0.87 (0.71-1.06) | 0.98 (0.76-1.26) | 1.68 (0.88-3.18) | 1.7 (0.73-3.99) |  |
| PCB 183 | Continuous^d^ | 0.7 (0.54-0.9)** | 0.87 (0.65-1.16) | 3.17 (1.35-7.44)** | 2.11 (0.76-5.83) |  |
|  | Q1 (<5.12) | 1 | 1 | 1 | 1 |  |
|  | Q2 (5.12-7.55) | 0.89 (0.73-1.09) | 0.95 (0.77-1.17) | 1.63 (0.77-3.46) | 1.38 (0.63-3.05) |  |
|  | Q3 (7.56-10.55) | 0.81 (0.66-1)* | 0.92 (0.74-1.14) | 1.83 (0.87-3.82) | 1.51 (0.68-3.34) |  |
|  | Q4 (>10.56) | 0.77 (0.63-0.95)* | 0.92 (0.73-1.16) | 2.44 (1.2-4.96)* | 1.66 (0.73-3.77) |  |
| PCB 187 | Continuous^d^ | 0.73 (0.55-0.96)* | 0.87 (0.62-1.23) | 2.94 (1.12-7.71)* | 2.23 (0.65-7.63) |  |
|  | Q1 (<12.66) | 1 | 1 | 1 | 1 |  |
|  | Q2 (12.66-18.55) | 0.82 (0.67-1) | 0.91 (0.73-1.13) | 1.5 (0.72-3.13) | 1.3 (0.59-2.85) |  |
|  | Q3 (18.56-25.81) | 0.87 (0.71-1.06) | 0.96 (0.78-1.2) | 1.68 (0.82-3.45) | 1.47 (0.67-3.27) |  |
|  | Q4 (>25.82) | 0.79 (0.64-0.97)* | 0.89 (0.7-1.13) | 2.15 (1.07-4.31)* | 1.89 (0.81-4.42) |  |

^a^Discrete-time Cox regression

^b^Logistic regression

^c^Adjusted with age, pre-pregnancy use of combined oral contraceptives, parity, maternal BMI, smoking, regularity of menses

^d^Log transformed

* p<0.05, **p<0.01

Excluded due to language barrier, not a resident in the county, beyond week 22 in their pregnancy and moving outside the study area

N=675

Blood collected where POPs and cotinine levels measured; Questionnaires answered

Excluded due to incomplete data

N=1764

Complete data on TTP, serum POP concentrations, age, parity, preventive method, regularity of menses, BMI and lifestyle factors

N=818

Invited pregnant women

N=7719

Women consented to participate

N=2582

Registered women in week 10 of pregnancy in Värmland, Sweden

Oct 2007 – March 2010

N=8394

Stratification according to age

(29 year old cutoff) and COC use

Individual chemicals analyzed using discrete-time Cox regression models (estimating FR, where FR > 1 denotes higher fecundability and shorter TTP, and FR < 1 denotes lower fecundability, and longer TTP) and logistic regression (estimating OR, where OR > 1 denotes higher odds for infertility and OR < 1 denotes lower odds for infertility)

Mixture of chemicals analyzed using WQS; chemicals of concern identified as those with weights more than 7.6%

**Supp Figure 1. Flowchart of recruitment process and analyses in the SELMA study.** Abbreviations: COC – combined oral contraceptives; FR – fecundability ratio; OR – odds ratio; POPs – persistent organic pollutants; TTP – time-to-pregnancy; WQS – weighted quantile sum


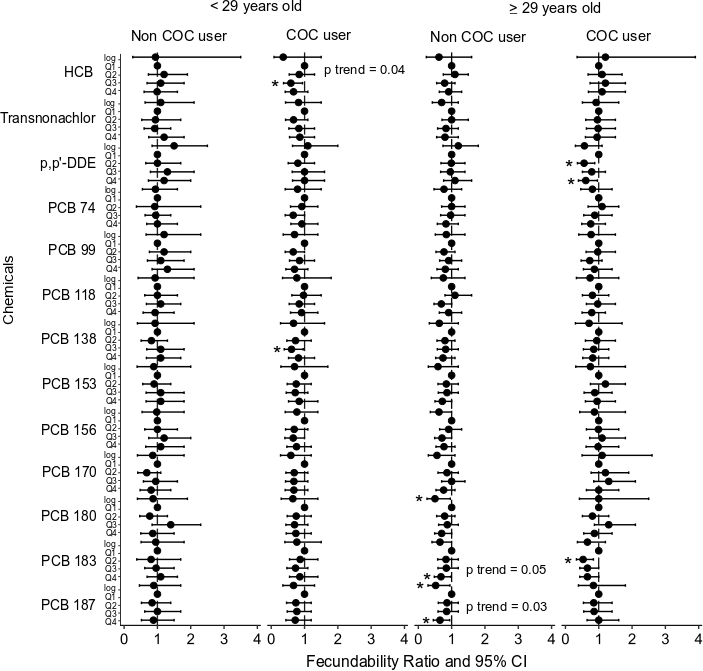


**Supp Fig 2. Sensitivity analyses with censoring at 10 months.** Dot shows fecundability ratio (FR) with 95% confidence interval error bars of chemicals as continuous (log) and quartiles (Q1 as reference). FR > 1 denotes higher fecundability and shorter TTP while FR < 1 denotes lower fecundability and longer TTP. FR indicated with * for p value < 0.05. P trend shown for significant linear trend of FR. Abbreviations: COC – combined oral contraceptives; HCB - hexachlorobenzene; p,p´-DDE – dichlorodiphenyldichloroethylene; PCB – polychlorinated biphenyls; Q1 – first quartile, Q2 – second quartile, Q3 – third quartile, Q4 – fourth quartile.


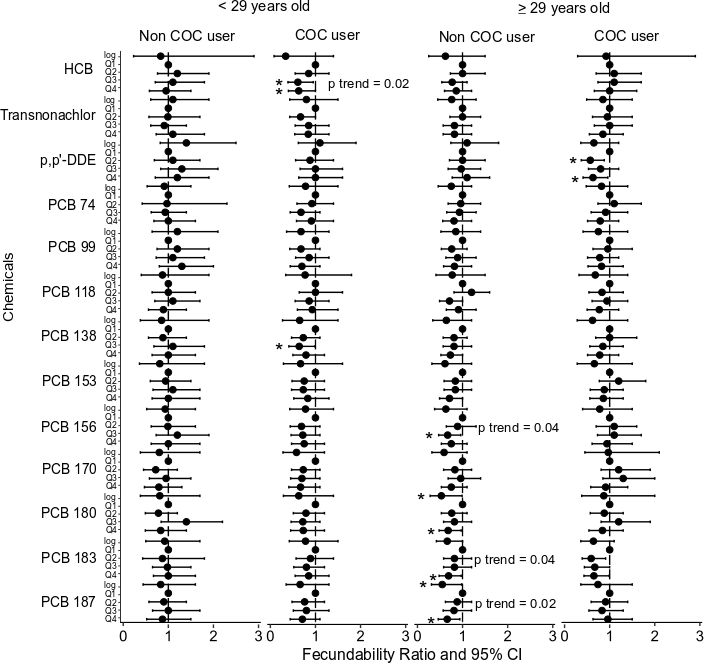


**Supp Fig 3. Sensitivity analyses with censoring at 14 months.** Dot shows fecundability ratio (FR) with 95% confidence interval error bars of chemicals as continuous (log) and quartiles (Q1 as reference). FR > 1 denotes higher fecundability and shorter TTP while FR < 1 denotes lower fecundability and longer TTP. FR indicated with * for p value < 0.05. P trend shown for significant linear trend of FR. Abbreviations: COC – combined oral contraceptives; HCB - hexachlorobenzene; p,p´-DDE – dichlorodiphenyldichloroethylene; PCB – polychlorinated biphenyls; Q1 – first quartile, Q2 – second quartile, Q3 – third quartile, Q4 – fourth quartile.
